# Supplementary figures and images for: Sex and organ specific proteomic responses to vitamin C deficiency in the brain, heart, liver, and spleen of Gulo-/- mice
Source: PLoS One. 2024 Oct 10;19(10):e0311857. doi: 10.1371/journal.pone.0311857 (PMC11476689; doi:10.1371/journal.pone.0311857)

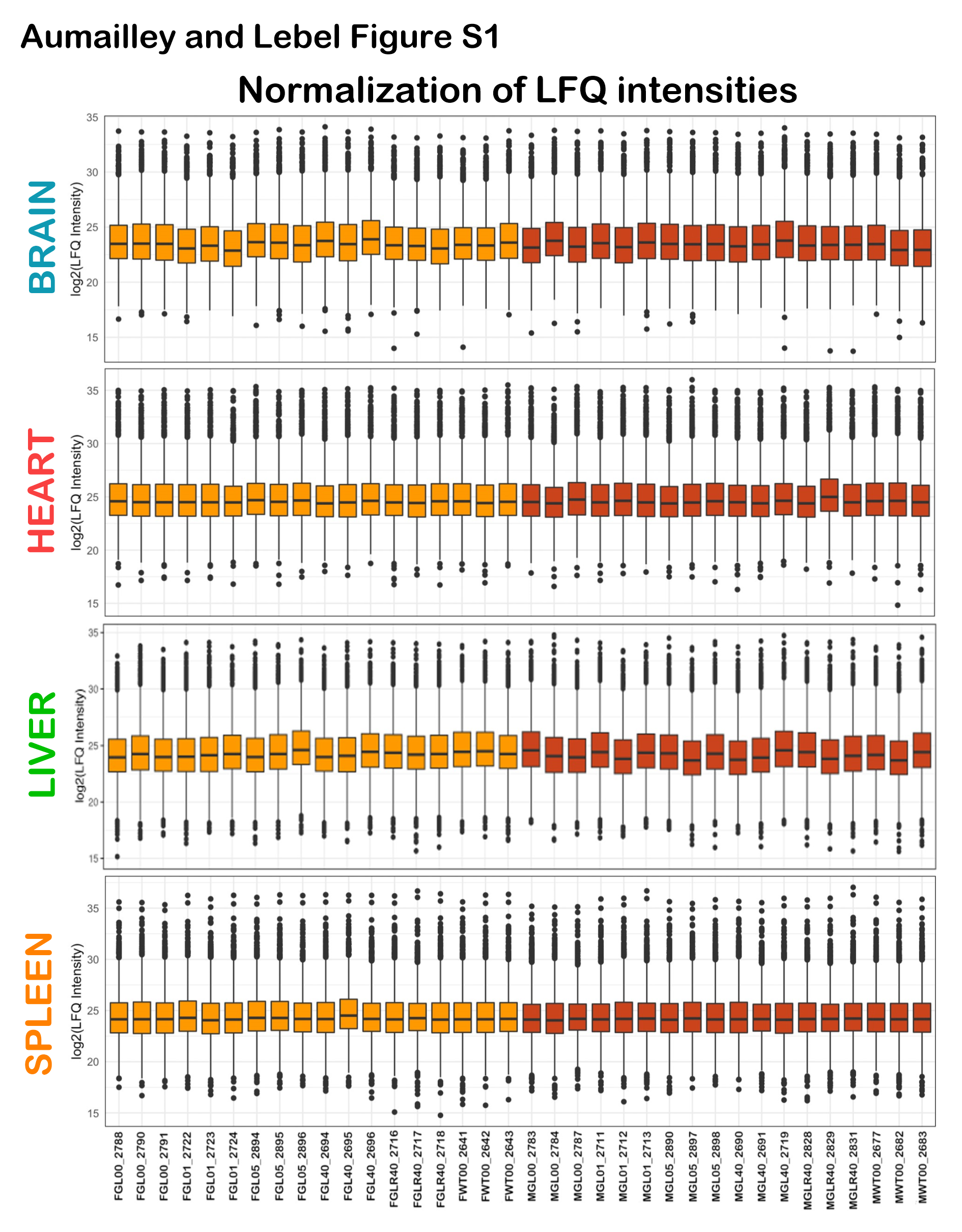

Supplement: S1 Fig — Box plot depicting the distribution of each individual sample after normalization of the LFQ intensities using MaxQuant (N = 18 females and N = 18 males) in each organ. (TIF) [file pone.0311857.s001.tif]

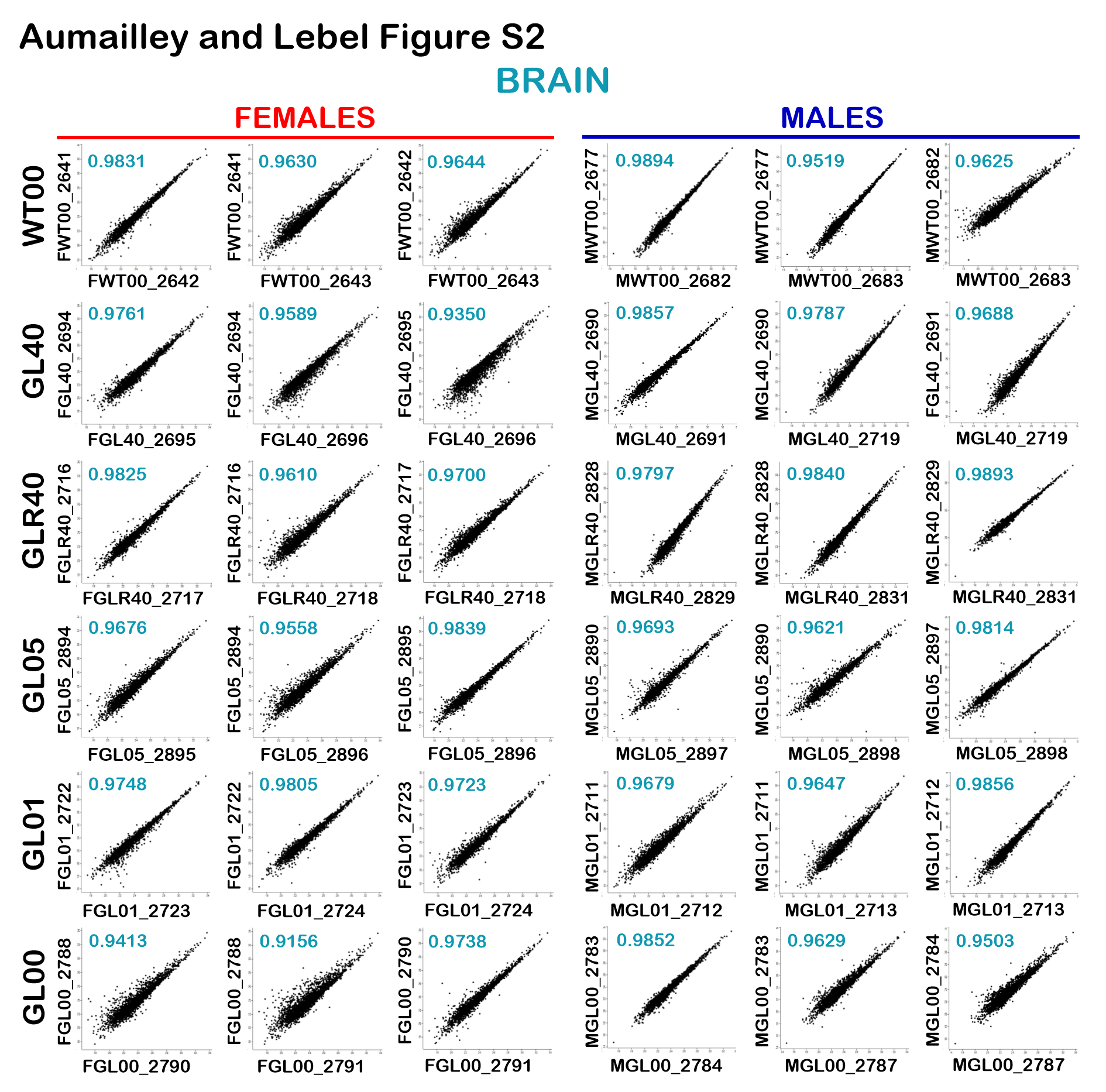

Supplement: S2 Fig — Pearson correlation coefficients obtained for each two-by-two comparison are indicated in blue on each graph. (TIF) [file pone.0311857.s002.tif]

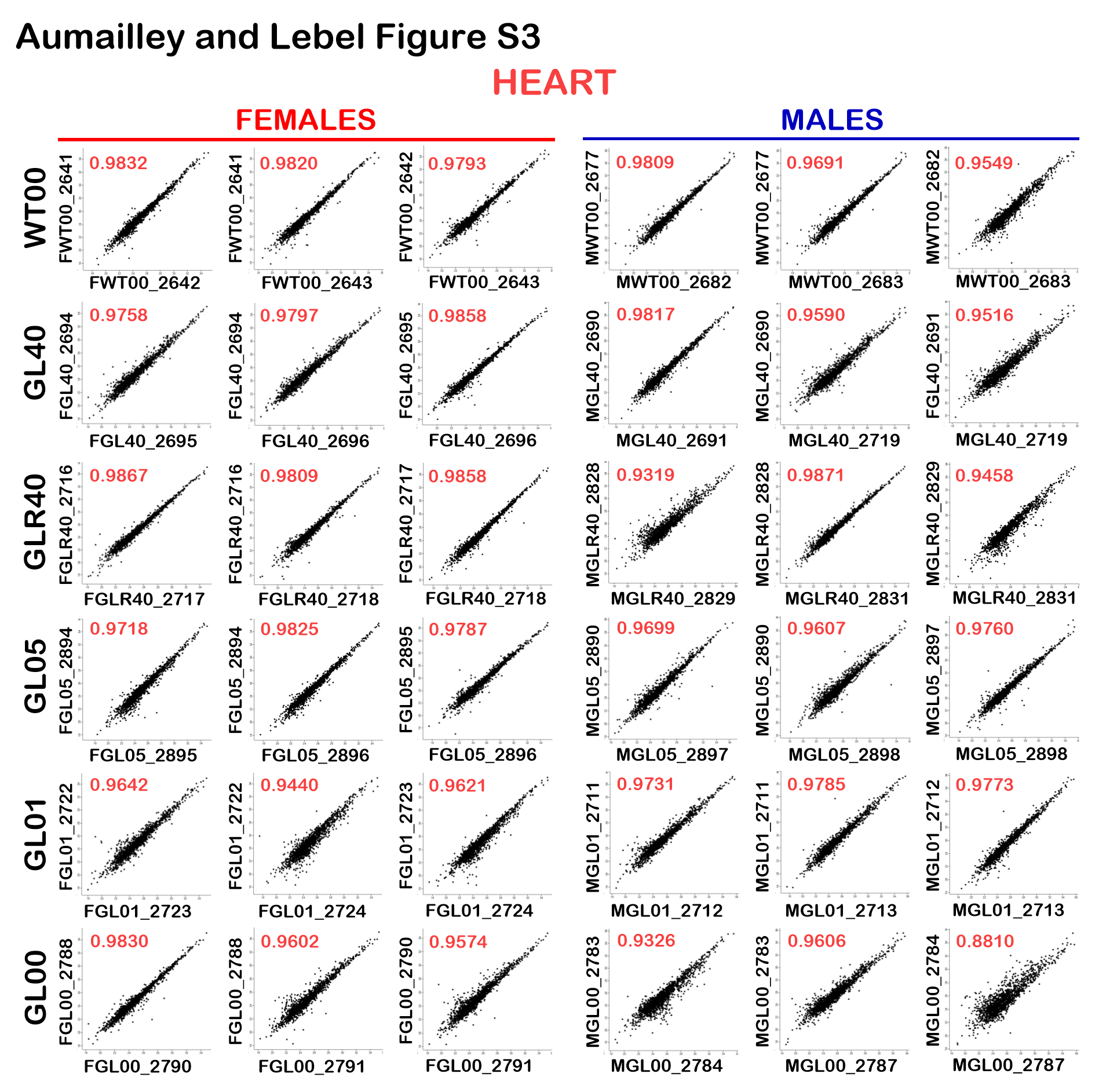

Supplement: S3 Fig — Pearson correlation coefficients obtained for each two-by-two comparison are indicated in red on each graph. (TIF) [file pone.0311857.s003.tif]

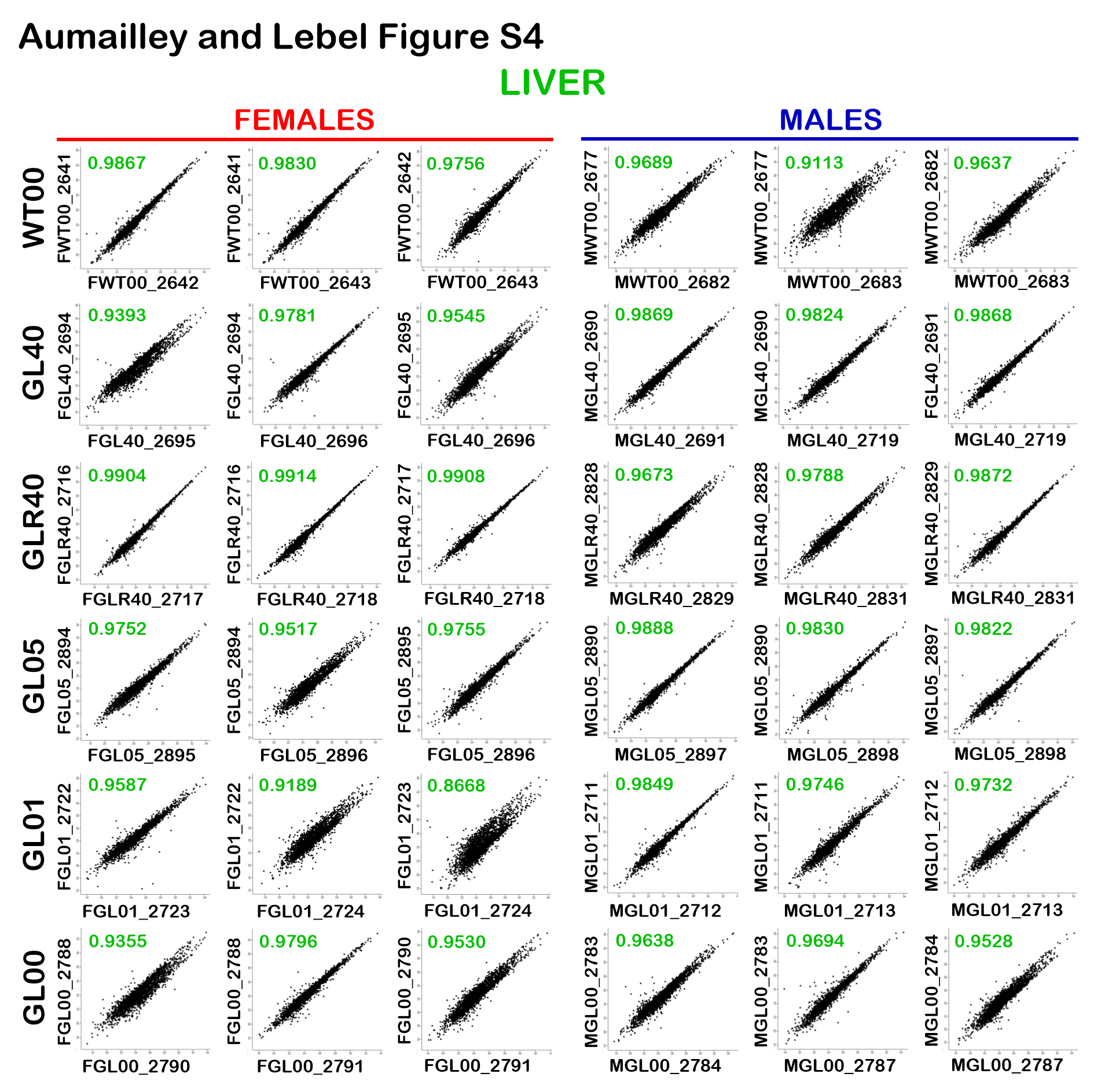

Supplement: S4 Fig — Pearson correlation coefficients obtained for each two-by-two comparison are indicated in green on each graph. (TIF) [file pone.0311857.s004.tif]

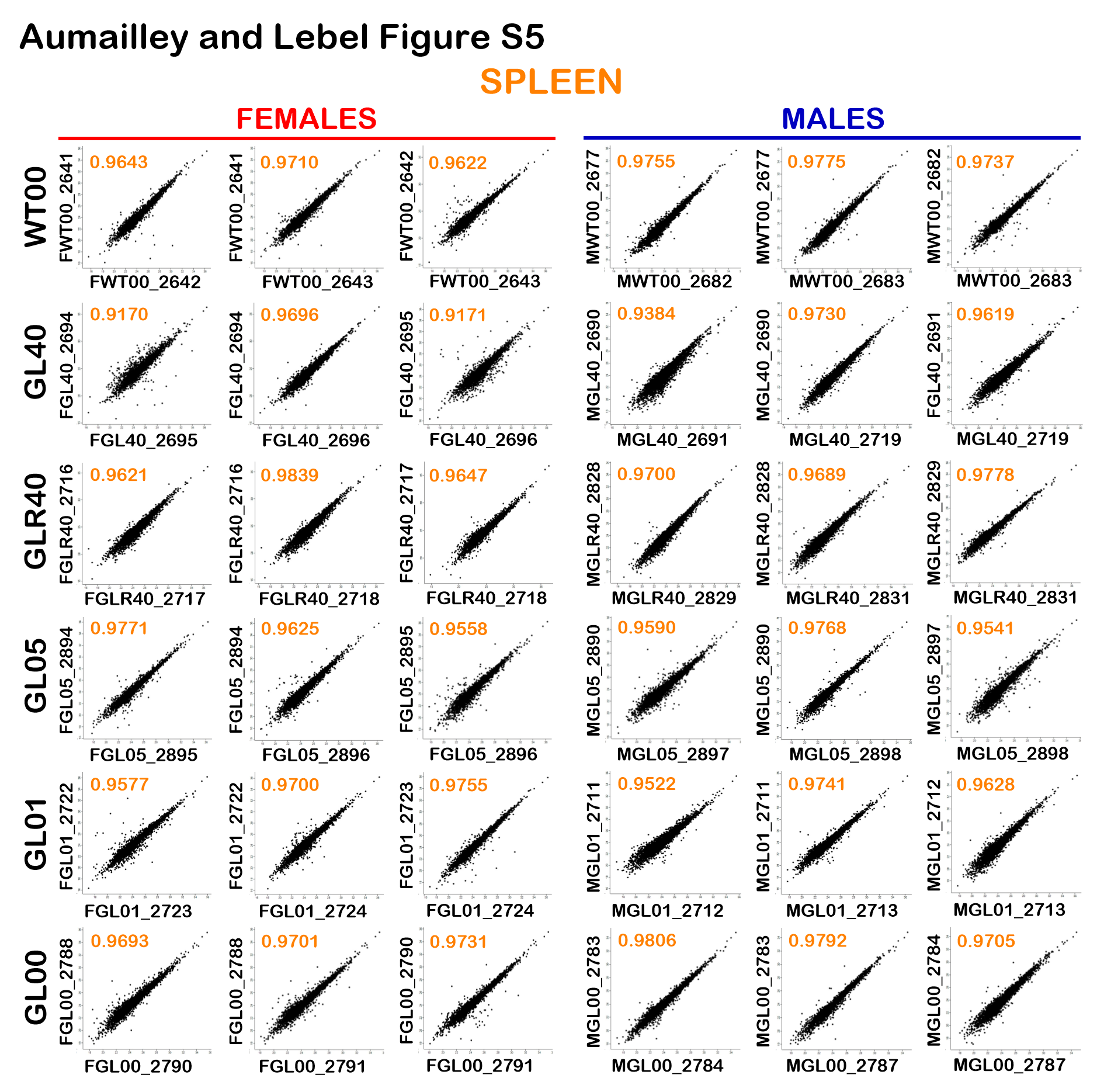

Supplement: S5 Fig — Pearson correlation coefficients obtained for each two-by-two comparison are indicated in orange on each graph. (TIF) [file pone.0311857.s005.tif]

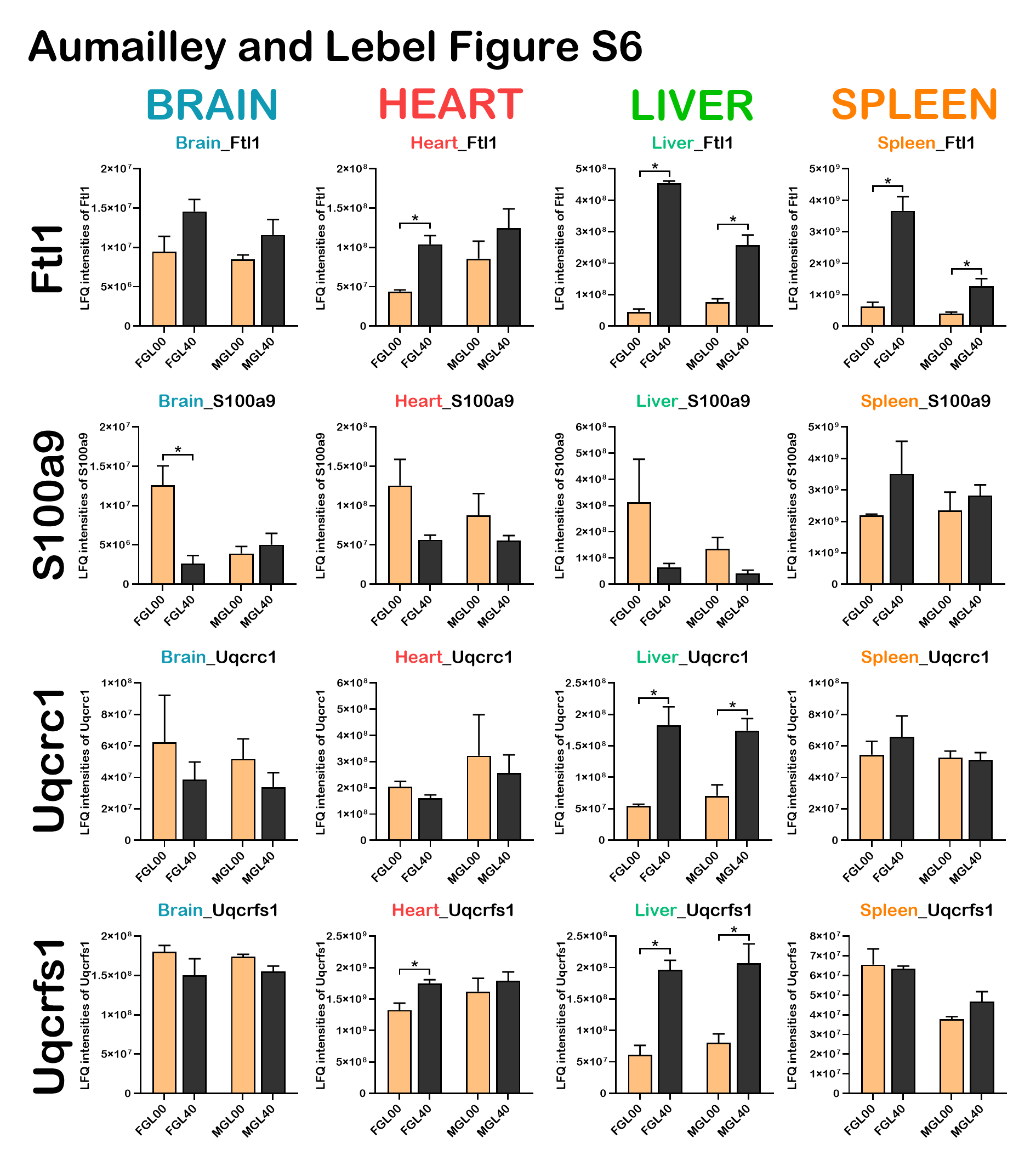

Supplement: S6 Fig — The histograms present the LFQ data for Ftl1, S100a9, Uqcrc1, and Uqcrfs1 proteins in the four different tissue lysates (N = 3 females and 3 males for each cohort; * p-value < 0.05; Welch’s t-test). (TIF) [file pone.0311857.s006.tif]

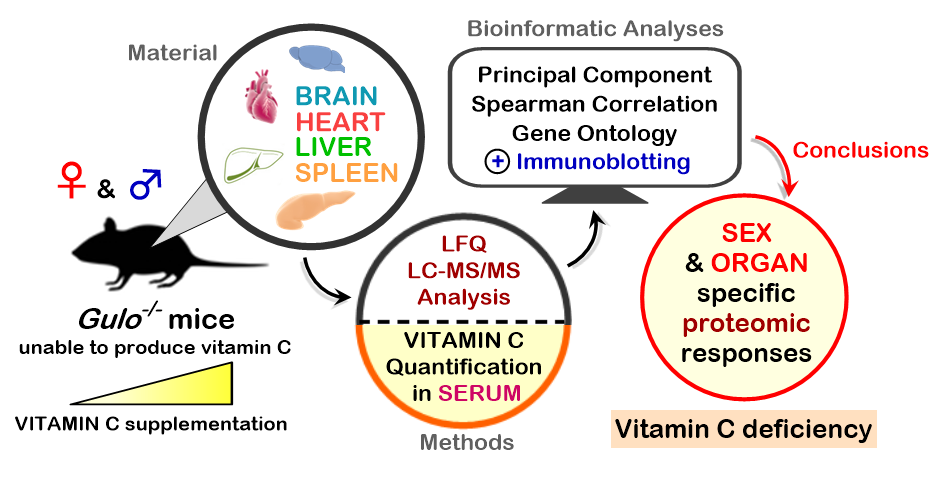

Supplement: S1 Graphical abstract — (TIF) [file pone.0311857.s020.tif]
