# Supplementary material for: Sex and organ specific proteomic responses to vitamin C deficiency in the brain, heart, liver, and spleen of Gulo-/- mice
Source: PLoS One. 2024 Oct 10;19(10):e0311857. doi: 10.1371/journal.pone.0311857 (PMC11476689; doi:10.1371/journal.pone.0311857)
Supplement: S1 Raw image — (PDF) [file pone.0311857.s019.pdf]

BRAIN

Ftl1

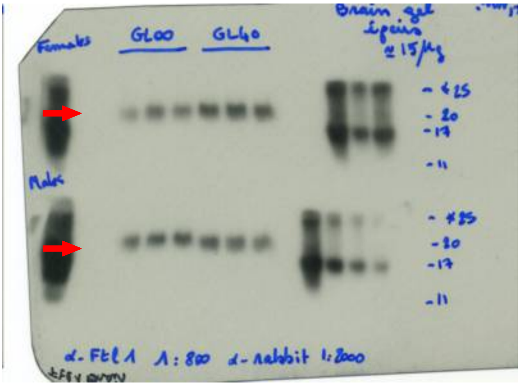

S100a9

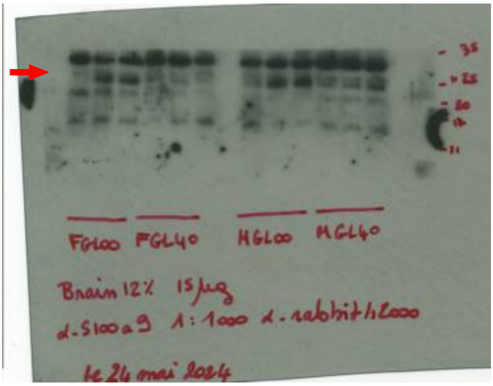

Uqcrc1

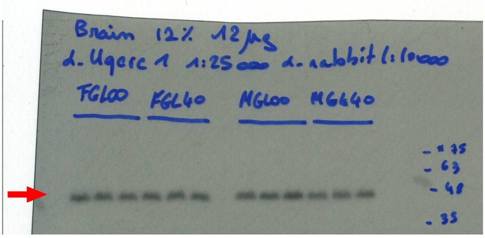

Uqcrfs1

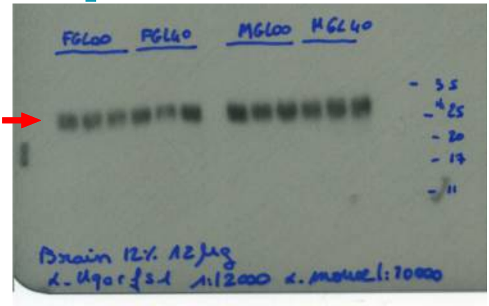

Coomassie

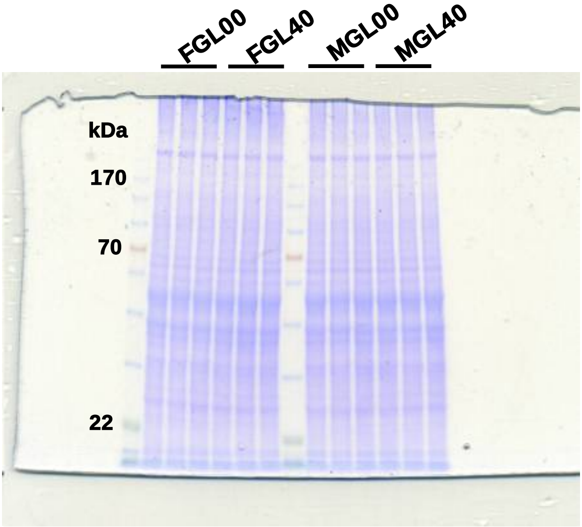

→ : **specific band**

Note : Other unpointed bands on the films are non-specified bands (or background from protein ladder marker)

HEART

Ftl1

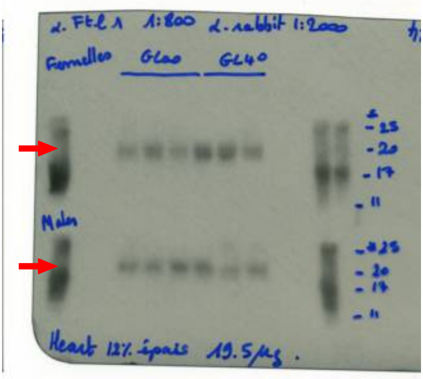

S100a9

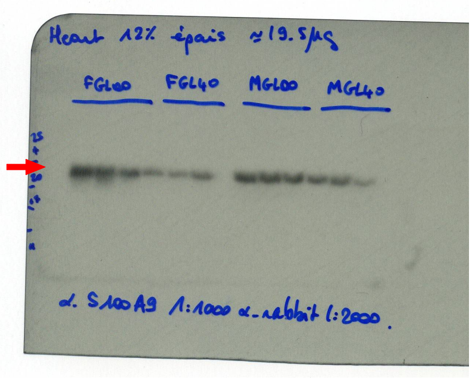

Uqcrc1

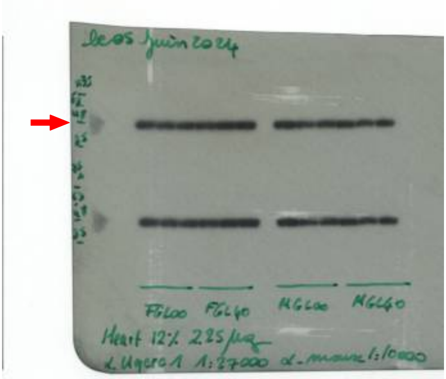

Uqcrfs1

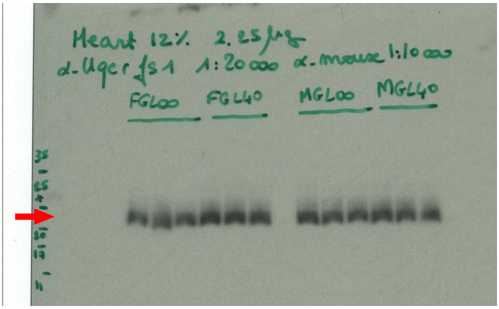

Coomassie

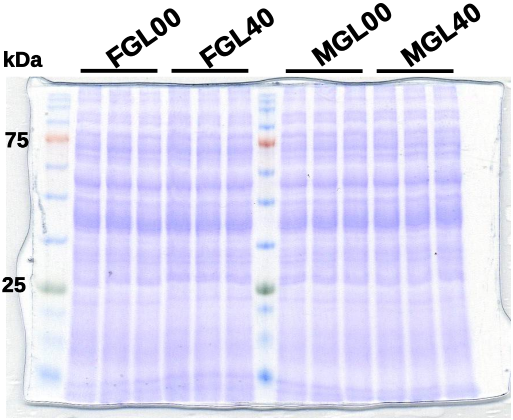

→ : specific band

Note : Other unpointed bands on the films are non-specified bands (or background from protein ladder marker)

LIVER

Ftl1

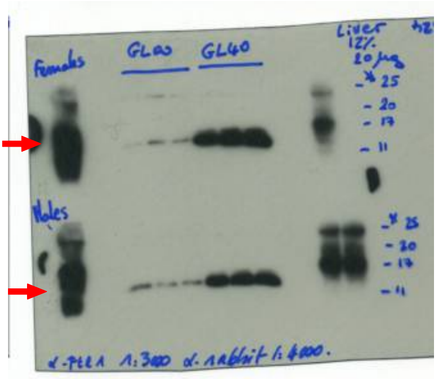

S100a9

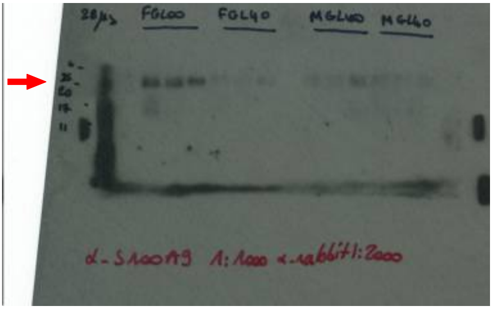

Uqcrc1

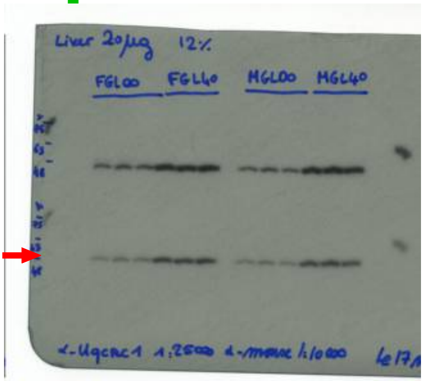

Uqcrfs1

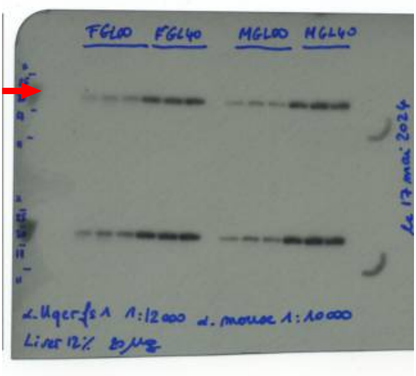

Coomassie

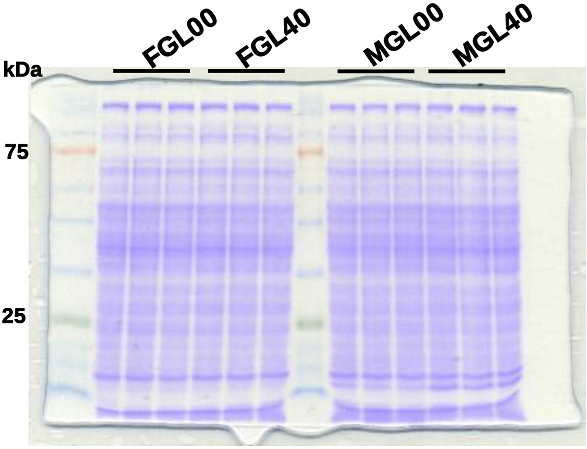

→ : **specific band**

Note : Other unpointed bands on the films are non-specified bands (or background from protein ladder marker)

SPLEEN

Ftl1

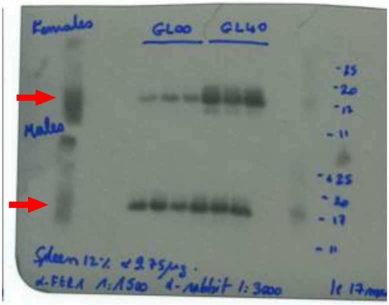

S100a9

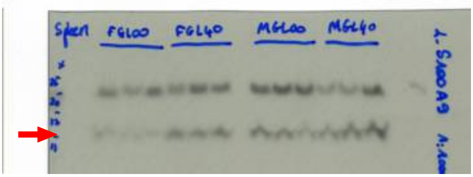

Uqcrc1

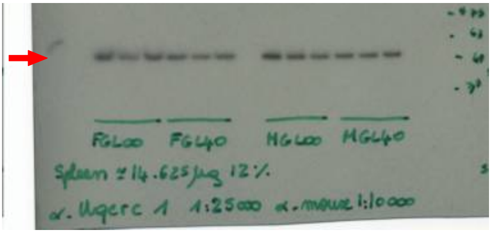

Uqcrfs1

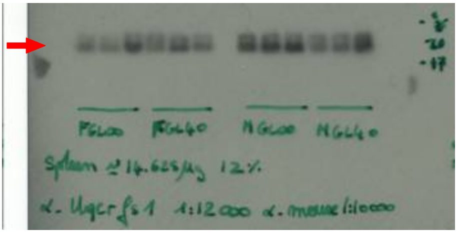

Coomassie

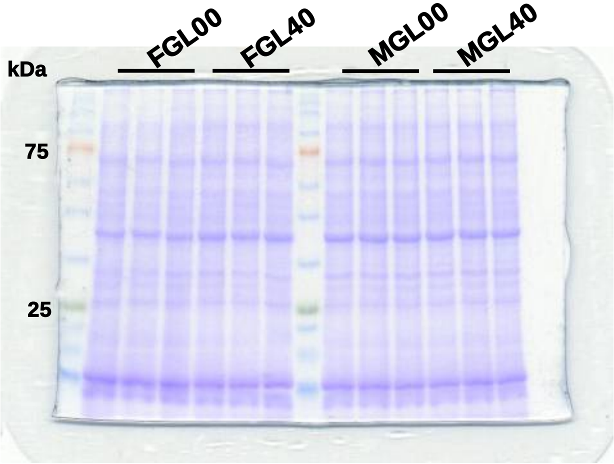

→ : specific band

Note : Other unpointed bands on the films are non-specified bands (or background from protein ladder marker)

SERUM

Ftl1

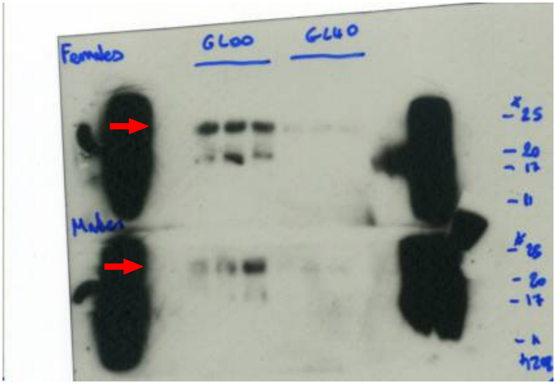

S100a9

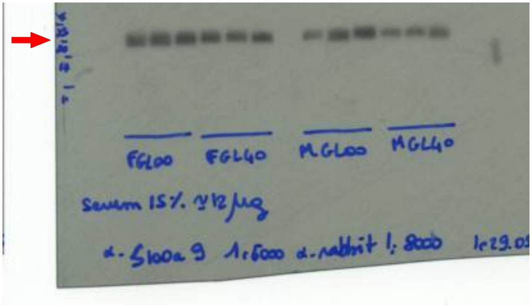

Coomassie

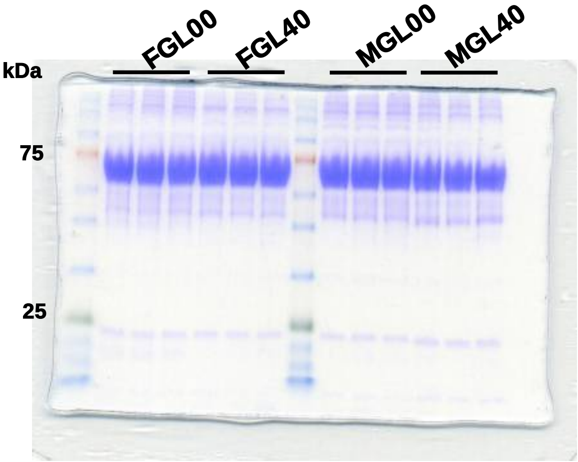

→ : specific band

Note : Other unpointed bands on the films are non-specified bands (or background from protein ladder marker)
