# Supplementary material for: Sex and organ specific proteomic responses to vitamin C deficiency in the brain, heart, liver, and spleen of Gulo-/- mice
Source: PLoS One. 2024 Oct 10;19(10):e0311857. doi: 10.1371/journal.pone.0311857 (PMC11476689; doi:10.1371/journal.pone.0311857)
Supplement: Attachment — Submitted filename: Response to Reviewers.docx [file pone.0311857.s023.docx]

September 24^th^, 2024

Dr. Swaroop Kumar Pandey

Academic Editor

PLOS ONE

Dear Dr. Swaroop Kumar Pandey,

Please find enclosed our revised manuscript entitled “Sex and organ specific proteomic responses to vitamin C deficiency in the brain, heart, liver, and spleen of *Gulo^-/-^* mice” by Lucie Aumailley and Michel Lebel. Both authors agree with the content of the revised version of the manuscript.

We are very grateful for the suggestions made by the referees. We agree that the work has been appreciably improved by virtue of their thoughtful comments. We, therefore, are returning a revised version of the manuscript to you with detailed response to the Reviewer #1 specific comment below and have revised the manuscript accordingly.

1) We also made changes in the manuscript to meet the journal’s style requirements.

2) As requested, we added the original blot/gel image data in the supporting information as a pdf file entitled “S1_Raw_Images”. This information is mentioned in the Materials and Methods section.

3) Funding Statement: This work was supported by the Canadian Institutes of Health Research, Canada (PJT-173398) to Michel Lebel.

4) Role of the funder: The funders had no role in study design, data collection and analysis, decision to publish, or preparation of the manuscript.

We hope our revised manuscript incorporating these changes will now be acceptable for publication in the journal **PLOS ONE**. Should you have any question about this manuscript, please do not hesitate to contact me. We look forward to hearing from you at your earliest convenience.

Yours sincerely,

Michel Lebel

Michel Lebel, PhD

Full professor Laval University

Centre de Recherche du CHU de Québec

Centre Hospitalier de l’Université Laval (CHUL)

2705, boul. Laurier, Local R-2714

Québec, Québec G1V 4G2

Canada

Tel: 418-525-4444 ext.46234

michel.lebel@crchudequebec.ulaval.ca

**Reviewer #1**

**Reviewer’s Comments**

The recent manuscript describes the differential protein expression in different tissues of male and female mice with the correlation of vitamin C concentration. The study concludes that the Proteome profile to decreasing serum vitamin C level is not only sex-dependent but also tissue specific. The manuscript is well written, results are clearly presented, and also recommended for the publication.

**Specific comment:**

The biological process which are similar in male and female are also showing different correlation with the vitamin C, explain the probable reason behind the same.

**Response:** As requested by reviewer #1, we explained in the discussion section the reason why the similar biological process correlated in opposite direction between females and males upon vitamin C deficiency. We added the following information in the text and introduced an additional reference.

GO analyses on the identified proteins correlating with serum vitamin C levels in the liver showed more biological processes modulated by vitamin C in females than in males. Interestingly, the hepatic tissue of *Gulo^-/-^* females exhibited a positive correlation between serum vitamin C and various proteins mainly involved in lipogenesis. In contrast, the liver of *Gulo^-/-^* males showed an inverse correlation between serum vitamin C and several proteins associated with lipid or fatty acid catabolism. Thus, fatty acid metabolic process (GO:0006631) has been significantly enriched in both females and males in the liver but showed different correlation with the vitamin C between the two sexes. Detailed examination of the proteins associated with this biological process revealed that the list of proteins in females (16 total) and the one from males (12 total) are different. This divergence probably explains the observed opposite correlation for fatty acid metabolic process in females and in males. Nevertheless, the overall outcomes of the regulation of these various proteins related to fatty acid metabolism tend toward a potential decreased accumulation of lipids under a vitamin C deficient condition in both females and males. Accordingly, a recent proteomic study indicated that sex hormone signaling plays a key role in lipid metabolism pathways and induces sexually dimorphic changes in the mouse liver proteome with a similar phenotypic outcome [35].

The new reference 35 is as follows:

35.	Harney DJ, Cielesh M, Roberts GE, Vila IK, Viengkhou B, Hofer MJ, et al. Dietary restriction induces a sexually dimorphic type I interferon response in mice with gene-environment interactions. Cell Rep. 2023;42(6):112559. Epub 2023/05/27. doi: 10.1016/j.celrep.2023.112559. PubMed PMID: 37243595.

**Reviewer #2**

Authors have presented a study where they have found out that Veitamin C concentrations in male and female mice differentially modulates the abundance of various proteins in hepatic tissues. To support their study, the authors have performed LC-MS/MS to identify and quantify proteins that correlate with serum vitamin C concentrations in thewhole brain, heart, liver, and spleen tissues in mice deficient for the enzyme LGulonolactone oxidase required for vitamin C synthesis in mammals. Their work shows that Vitamin C deficiency affect numerous biological processes that are tissue and sex specific dependent. Their study provides a preliminary comprehensive analysis of various proteins that significantly correlate with Vitamin C levels. This information can be useful for the Science community and can add to some therapeutic advancements in the longer run.

**Response:** Thank you.
