# Supplementary material for: Sex and organ specific proteomic responses to vitamin C deficiency in the brain, heart, liver, and spleen of Gulo-/- mice
Source: PLoS One. 2024 Oct 10;19(10):e0311857. doi: 10.1371/journal.pone.0311857 (PMC11476689; doi:10.1371/journal.pone.0311857)
Supplement: Attachment — Submitted filename: PLoS reviewer comment.docx [file pone.0311857.s021.docx]

**Reviewer’s Comments**

The recent manuscript describes the differential protein expression in different tissues of male and female mice with the correlation of vitamin C concentration. The study conclude that the the Proteome profile to decreasing serum vitamin C level is not only sex-dependent but also tissue specific. The manuscript is well written, and results are clearly presented and also recommended for the publication*.*

**Specific comment:**

The biological process which are similar in male and female are also showing different correlation with the vitamin C, explain the probable reason behind the same.
