# Supplementary material for: Sex and organ specific proteomic responses to vitamin C deficiency in the brain, heart, liver, and spleen of Gulo-/- mice
Source: PLoS One. 2024 Oct 10;19(10):e0311857. doi: 10.1371/journal.pone.0311857 (PMC11476689; doi:10.1371/journal.pone.0311857)
Supplement: Attachment — Submitted filename: Reviewer comments.docx [file pone.0311857.s022.docx]

Authors have presented a study where they have found out that Veitamin C concentrations in male and female mice differentially modulates the abundance of various proteins in hepatic tissues. To support their study, the authors have performed LC-MS/MS to identify and quantify proteins that correlate with serum vitamin C concentrations in thewhole brain, heart, liver, and spleen tissues in mice deficient for the enzyme LGulonolactone oxidase required for vitamin C synthesis in mammals. Their work shows that Vitamin C deficiency affect numerous biological processes that are tissue and sex specific dependent. Their study provides a preliminary comprehensive analysis of various proteins that significantly correlate with Vitamin C levels. This information can be useful for the Science community and can add to some therapeutic advancements in the longer run.
